# The Role of Human Milk Oligosaccharides in Myelination, Socio-Emotional and Language Development: Observational Data from Breast-Fed Infants in the United States of America

**DOI:** 10.3390/nu15214624

**Published:** 2023-10-31

**Authors:** Purva Rajhans, Fabio Mainardi, Sean Austin, Norbert Sprenger, Sean Deoni, Jonas Hauser, Nora Schneider

**Affiliations:** 1 Brain Health Department, Nestlé Institute of Health Sciences, Nestlé Research, Société des Produits Nestlé S.A., Vers-chez-les-Blanc, 1000 Lausanne, Switzerland; jonas.hauser@rdls.nestle.com (J.H.); nora.schneider@rdls.nestle.com (N.S.); 2 Department of Data Sciences & Precision Nutrition, Nestlé Institute of Health Sciences, Nestlé Research, Société des Produits Nestlé S.A., Vers-chez-les-Blanc, 1000 Lausanne, Switzerland; fabio.mainardi@rd.nestle.com; 3 Nestlé Institute of Food Safety and Analytical Sciences, Nestlé Research, Société des Produits Nestlé S.A., Vers-chez-les-Blanc, 1000 Lausanne, Switzerland; sean.austin@rdls.nestle.com; 4 Gastro-Intestinal Health Department, Nestlé Institute of Health Sciences, Nestlé Research, Société des Produits Nestlé S.A., Vers-chez-les-Blanc, 1000 Lausanne, Switzerland; norbert.sprenger@rdls.nestle.com; 5 Advanced Baby Imaging Lab, Rhode Island Hospital, Providence, RI 02912, USA; sdeoni@mac.com; 6 Department of Radiology, Warren Alpert Medical School at Brown University, Providence, RI 02903, USA; 7 Spinn Neuroscience, Seattle, WA 98275, USA

**Keywords:** human milk oligosaccharides (HMOs), myelination, social-emotional development, language development

## Abstract

Infancy is a critical period for neurodevelopment, which includes myelination, synaptogenesis, synaptic pruning, and the development of motor, social-emotional, and cognitive functions. Human milk provides essential nutrients to the infant’s developing brain, especially during the first postnatal months. Human milk oligosaccharides (HMOs) are a major component of human milk, and there is growing evidence of the association of individual HMOs with cognitive development in early life. However, to our knowledge, no study has explained these associations with a mechanism of action. Here, we investigated possible mediating associations between HMOs in human milk, brain myelination (measured via myelin water fraction), and measures of motor, language (collected via the Bayley Scales of Infant and Toddler Development (Bayley-III)), and socioemotional development (collected via the Ages and Stages Questionnaire: Social-Emotional Version (ASQ-SE)) in healthy term-born breast-fed infants. The results revealed an association between 6′Sialyllactose and social skills that was mediated by myelination. Furthermore, associations of fucosylated HMOs with language outcomes were observed that were not mediated by myelination. These observations indicate the roles of specific HMOs in neurodevelopment and associated functional outcomes, such as social-emotional function and language development.

## 1. Introduction

Infancy is a sensitive period for rapid brain maturation and corresponds with the emergence of sensorimotor, language, and social-emotional skills and abilities [1] that form a strong foundation for life-long learning and development. Early infant nutrition, particularly early predominant or exclusive breastfeeding, is an important factor positively associated with brain development in general, [2,3,4] and specifically with neurodevelopmental processes, such as brain myelination related to cognitive, intellectual, social, and behavioral development [2,3,4,5,6,7]. In line with the WHO guidance, human milk is the exclusive and recommended nutrition during the first 6 months of an infant’s life, providing nutrients and bioactive components. Furthermore, these nutrients and bio-active components of human milk support neurodevelopmental processes of an infant’s developing brain including myelination, synaptogenesis, and synaptic pruning [8].

Among human milk components, human milk oligosaccharides (HMOs) represent the third most abundant and solid component after lipids and lactose. Known for their structural diversity, HMOs can be classified according to their monosaccharide composition as neutral, fucosylated (when containing fucose), or sialylated (when containing a sialic acid). HMOs have been proposed to affect multiple physiological and biological functions [9], including the development of immune, gastrointestinal, and central nervous systems [10,11,12]. Several basic research models showed HMO(s) supplementation, or selective removal from mouse milk by genetic means, to affect neurodevelopment and cognitive outcomes [13,14,15,16,17]. Recent observational data with breastfed infants suggest 2′Fucosyllactose (2′FL) is associated with synapse development [18,19] at 1 month of age and later cognitive and motor scores at 6 months and older [15,18]. Furthermore, 3′Sialyllactose (3′SL) has been associated with increased structural connectivity in the infant brain at 1 month and language scores at 12 months [18]. Similarly, another study also reported an association between 3′SL in human milk and infant language development [20]. The second type of sialyllactose in human milk, 6′Sialyllactose (6′SL) has been associated with infant motor and overall cognitive scores at 18 months [21]. Additionally, the non-sialylated and non-fucosylated Lacto-N-tetraose (LNT) has been associated with improved social skills in 1-year-old infants [22]. Lastly, there is evidence from a recent publication linking generally fucosylated HMOs to executive functions in toddlerhood [23]. Collectively, the currently available data suggest that some HMOs may be linked with brain and cognitive development, yet consistency among the different studies is limited. Equally, knowledge of possible affected brain processes and mechanisms of action have not been explored.

Recent work started to investigate links between HMOs and structural connectivity of the brain, a process that is strongly dependent on sialic acid-containing glycoproteins and glycolipids [19]. Brain myelination is another process highly dependent on sialic acid bound to gangliosides, for example [24]. More specifically, Neu5Ac is one of the two sources of sialic acid that is received by the infant from maternal milk in the form of 6′SL. Furthermore, Neu5Ac is implicated in ganglioside formation which is, in turn, critical for myelination. Maturation of the myelinated white matter is a hallmark process of neurodevelopment in infants, critical for efficient brain function, and influenced by nutrition [7]. Recent years saw an increasing emphasis on linking changes in early childhood brain myelination with cognitive, behavioral, and social-emotional outcomes [7,25,26]. Established associations between myelination and emerging language, motor, and social-emotional skills [27,28,29] offer a potential pathway by which HMOs may influence these skills. However, direct evidence linking HMOs, myelin, and behavior has not been established. Based on the malleability of myelination through nutrition and the expected actions of HMOs to (i) support the maturation of the gut and its microbiome, as well as (ii) provide building blocks or metabolic signals via sialic acid, we speculated that specific HMOs may affect functional maturation of the developing brain and that this would be reflected in the myelination pattern.

### The Current Study

The aim of the current study was to investigate the impact of HMO concentration in human milk during the first 4 months of age on measures of motor, language, and social-emotional development in typically developing, term-born breastfed infants and to explore the role of myelination in that relation.

## 2. Materials and Methods

### 2.1. Study Population and Data

Infants for this study were enrolled as the non-randomized observational breastfeeding reference group of a prospective longitudinal randomized control trial, see Supplementary Figure S2A,B (clinical trial registry with the identifier: NCT03111927). Results for the randomized arms were published previously [30]. Infants for the trial were recruited and followed over 24 months at 2 study sites in the United States of America (Rhode Island Hospital in Providence, RI, and Pennington Biomedical Research Center, in Baton Rouge, LA), with 8 study visits conducted at V0 (2–5 weeks), V1 (6 ± 1 week of life), V2 (3 months ± 2 weeks), V3 (6 months ± 2 weeks), V4 (9 months ± 2 weeks), V5 (12 months ± 2 weeks), V6 (18 months ± 3 weeks), and V7 (24 months± 4 weeks). For the observational breastfeeding arm, mothers and their infants were recruited at 6 weeks following delivery and were included if more than 90% of nutritional intake was breast milk with no more than 10% of nutritional intake being infant formula or solids through at least 2 months of life. Recruitment occurred between May 2017 and March 2020 via self-referral, and maternal screenings were performed during the third trimester of pregnancy up to and including post-delivery. Following written informed consent (screening visit), sociodemographic information and medical and family histories were collected, as well as a physical and neurological examination of the infant. Withdrawal from the study was possible at any point and with no further evaluations or any additional data collection. The research ethics boards at both clinical sites approved the protocol.

Other specific inclusion criteria included English-speaking mothers at least 18 years of age with a maternal intelligence quotient (IQ, Wechsler Adult Intelligence Scale) of at least 70, healthy pregnancy with uncomplicated delivery at 38–41 weeks gestation, and birth weight >2000 g.

Exclusion criteria included the following: (a) delayed birth (>41 weeks + 6 days gestation) as reported in the medical record when available, (b) birth weight <2000 g or small for gestation age (birth weight less than the 10th percentile for the gestational age) or large for gestational age (weight, length, or head circumference that lies above the 90th percentile) as reported in medical record when available; use of anti-epileptic drugs, antidepressants, benzodiazepines, cytotoxic drugs, dopamine agonists, and/or opioids.

### 2.2. Human Milk Samples

Human milk samples were longitudinally collected from mothers at V0 (2–5 weeks), V1 (6 weeks), and V2 (3 months) study visits using a hospital-grade electric breast pump, and a full expression sample was collected. Samples were collected between 10 AM and 12 PM from the right breast. Mothers were asked to empty the right breast approximately 2 h prior to milk sampling.

### 2.3. Cognitive Assessments

Infant cognitive and social development was assessed using the Bayley Scales of Infant and Toddler Development, 3rd edition (BAYLEY-III) at V3, V5, and V7 corresponding to approximately 6, 12, and 24 months of age and the Ages and Stages Questionnaire: Social-Emotional, 2nd edition (ASQ-SE:2) at all visits from V2 to V7, corresponding to about 3, 6, 9, 12, 18, and 24 months of age.

### 2.4. Neuroimaging

Magnetic Resonance Imaging (MRI) scans were performed at visits V2, V3, V5, V6, and V7 corresponding to approximately 3, 6, 12, 18, and 24 months of age (Supplementary Figure S2B). Brain myelination was assessed using a myelin water magnetic resonance imaging technique (mcDESPOT) on a Siemens 3 Tesla MRI scanner [31]. All neuroimaging data were collected during natural non-sedated sleep [32]. From the mcDESPOT data, mean myelin water fraction (MWF) values were calculated throughout the brain in 176 regions of anatomical interest (hereafter indicated as independent components, or ICs).

MWF calculation was performed using a previously described analytical pipeline [33] that includes linear registration, skull-stripping of non-brain signals, calibration and correction of main and transmit magnetic field inhomogeneities, and finally calculation of MWF. Prior to analysis, all data were visually inspected for significant motion artifacts. The result MWF images were then non-linearly registered to a standard analysis space using the ANTs (v 2.2) registration tools. ROIs determined on the basis of longitudinal trajectories were then superimposed on the aligned images and mean MWF values were calculated for each and stored for subsequent statistical analysis.

### 2.5. Analytical Methods for Human Milk Quantification

HMOs (Supplementary Figure S1) were analyzed using ultra-high-performance liquid chromatography with fluorescence detection (UHPLC-FLD) according to the method of Austin and Benet [34]. 2′FL, 3FL, 3′SL, 6′SL, LNT, LNnT, and LNFP-I were quantified against genuine standards of analytical quality, all other HMOs were quantified against maltotriose assuming equimolar response factors.

Sialic acid was quantified using high-performance liquid chromatography with a fluorescence detector.

### 2.6. Statistical Analysis

We tested for associations between cognitive measures (BAYLEY-III and ASQ-SE:2) and possible confounding maternal and infant parameters: number of siblings, mode of delivery, maternal education, gestational age, sex, and child age. To account for non-normally distributed and skewed data, we applied Wilcoxon tests to compare scores between 2 groups (men vs. women, C-section vs. vaginal) and Spearman correlations to measure association with a numerical variable (age, number of siblings). We then used the significant associations to select the covariates in the analyses described hereafter.

Spearman correlations with a Benjamini–Hochberg correction to account for multiple testing were used to investigate the direct pairwise associations between 1. HMOs and cognitive measures (BAYLEY-III and ASQ-SE:2) and 2. HMOs and MWF measures. We analyzed the three BAYLEY-III subscales (cognition, language, and motor development) and the total ASQ-SE:2 score. For the significant correlations, we then fit linear models, adjusted for relevant covariates. In Equations (1) and (2), alpha is the intercept in the model. The other coefficients (beta, gamma, delta, eta) are the coefficients to be estimated in the model. Epsilon represents the error term. HMO stands for the concentration in mg/L.

(1) $${ASQ}_{}=\alpha +\beta HMO+\gamma VISIT+\delta HMO*VISIT+\eta Maternal.Education+\varepsilon \;$$

(2) $$MWF=\alpha +\beta \log\left(age\right)+\gamma HMO+\delta Maternal.Education+\varepsilon \;$$

These univariate analyses helped us to identify the time points and variables of interest, showing the strongest associations. We looked for associations that were consistent across time points. Similarly, we calculated Spearman correlation coefficients between breastmilk HMO concentration and MWF measures across each of the 176 anatomical ROIs, including delivery method, number of siblings, child age and biological sex, family income, and mother’s education as additional covariates.

Based on identified time points showing statistically significant associations (after correction for multiple testing), we then performed mediation analyses. The aim of a mediation analysis is to understand the underlying mechanism by which one variable influences another variable through a third (mediator) variable. This is performed by decomposing the total exposure–outcome effect into a direct effect and an indirect effect using the mediator variable. Mediation (or path models) can be estimated with a series of regression models. These regression models contain predictors that may cause changes in the outcome, and variables that may be causally prior to the outcome are used as predictors.

We used the ASQ-SE:2 at 12 months as the dependent variable, the 6′SL concentrations at various time points as independent variables, and the myelin water fraction at 3 months as the mediator. We used bootstrapped confidence intervals for the mediation/moderation effects. We report the proportion mediated, the total estimated effect, and the *p*-values estimated using bootstrapping. Quasi-Bayesian approximation was used for 95% confidence intervals, with 1000 Monte Carlo draws.

All analyses were performed with R, version 4.0.2. Mediation analysis was conducted with the R package mediation, version 4.5.0 [35].

## 3. Results

### 3.1. Population and Demographics

The mother/infant dyad cohort studied here was recruited as the non-randomized breastfed reference group of an intervention trial with two randomized formula-fed groups. The flow chart is depicted in Supplementary Figure S2A. A total of 293 mothers and 193 infants were screened for eligibility, of which 81 were randomized to formula (published previously [30]), 4 failed additional inclusion criteria, and 108 mother/infant dyads were enrolled in the breastfed infant/mother cohort studied here. Breast milk samples were available from a total of 107 mothers, with 106 samples available at V0, 97 at V1, and 76 at V2. 

Summary demographic information and characteristics for the mothers and infants are shown in Table 1.

### 3.2. HMOs

The sum of the measured HMOs decreased during the first 3 months of lactation from 9182 (2013) mg/L at V0 to 7887 mg/L (1813) at V1 and 6248 mg/L (1322) (mean (SD)) at V2.

**Figure 1:**
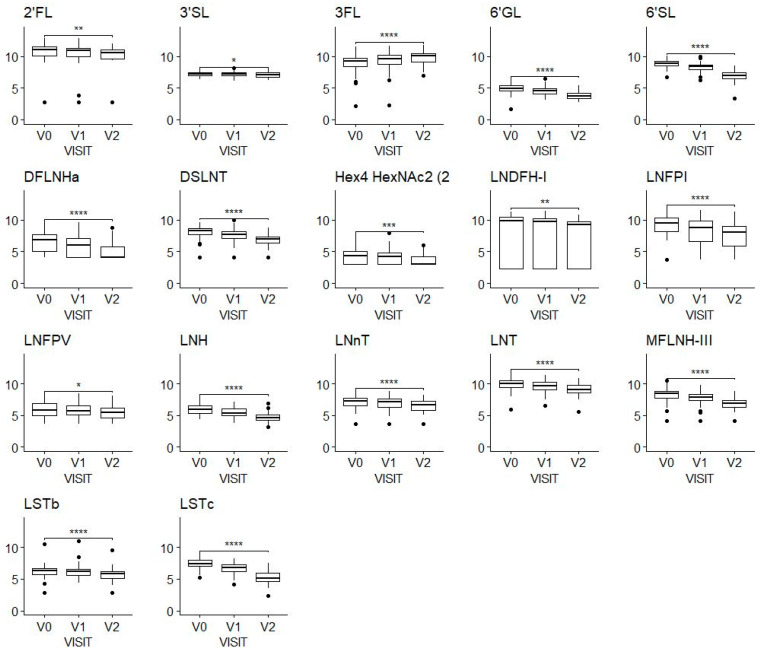
HMO concentrations at the three-time windows of lactation shown by visits V0 (2–5 weeks), V1 (6 ± 1 week) and V2 (3 months ± 1 week); values on the *y*-axis are log-transformed concentrations (mg/100 mL). A Wilcoxon test was applied to compare concentrations at V0 and V2. * Means *p*-value < 0.05, ** means *p*-value < 0.01, *** means *p*-value < 0.001, **** means *p*-value < 0.0001. The figure includes only HMOs with a significant difference. Sample sizes were N = 106 at V0, N = 97 at V1, N = 76 at V2.

We observed a decrease in almost all the HMOs over time (Figure 1). A noticeable exception to this general observation is 3FL, which showed an apparent increase from a mean of 704 mg/L (SD, 538 mg/L) at V0 to a mean of 1118 mg/L (SD, 698 mg/L) at V2.

### 3.3. Correlations between Human Milk HMO Concentration and ASQ-SE:2

Table 2 summarizes the ASQ-SE:2 scores at each time point.

Evaluation of possible confounding maternal and infant parameters revealed a significant negative correlation between maternal education and infant ASQ-SE:2 at 6 months (Spearman ρ=−0.3) and 24 months (ρ=−0.4). Hence, models were adjusted for maternal education.

To explore possible relations between HMOs and the development of social skills, we ran correlation models adjusted for possible confounding parameters. Linear models, as defined in Formula (1) in the Methods, were run for all the HMOs. The only significant association was found between ASQ:SE-2 at 12 months and the concentration of 6′-SL. The concentration of 6′SL was negatively associated with ASQ:SE-2 (β = −0.04, 95% CI =−0.06–−0.02), see Table 3. Spearman correlations at single time points between ASQ:SE-2 at 12 months and 6′SL were −0.43 at V0, −0.44 at V1, and −0.36 at V2, all significant (*p* < 0.01). Note that, since lower ASQ-SE:2 scores correspond to higher social skills, the negative correlations are suggestive of a beneficial effect of 6′SL.

Aligned with the 6′SL observation, ASQ:SE-2 scores at 12 months were also negatively correlated with the concentrations of total sialic acid at V0 (*ρ* = −0.38, *p* < 0.05) and at V1 (*ρ* = −0.39, *p* < 0.05). At V2, this association did not reach statistical significance, although we still saw a negative coefficient.

### 3.4. Correlations between Human Milk HMO Concentration and Myelination

With the hypothesis in mind that HMOs may play a role in the myelination processes, we next investigated associations between HMOs and MRI-measured brain myelination.

Summary statistics for myelin water fraction in the whole brain are reported in Supplementary Table S1. Two images were excluded from the analyses due to motion artifacts.

The child’s sex, maternal education, and delivery method were not associated with myelin water fraction. On the other hand, myelination is strongly dependent on age, even within the time window of a given study visit (Supplementary Figure S3).

After fitting age-adjusted linear models (Equation (2) in the Methods) to each HMO and each independent component, we did find several significant associations (Supplementary Table S2).

In particular, 6‘SL was significantly and positively associated with several ICs at 3 months, at multiple times of lactation: notably, IC67 (frontal lobe, internal capsule, thalamic radiations, temporal lobe, inferior longitudinal fasciculus, frontal pole Broca’s area, Brodman area 9) was associated with 6′SL at all time points (V0, V1, V2); IC150 (cingulate, insula, frontal orbital cortex, thalamus, caudate nucleus) was associated with 6′SL at V0, V1, and V2. Scatterplots for ICs 67 and 150 are shown in Supplementary Figure S4. These independent components were also positively correlated with total sialic acid.

### 3.5. Does Myelination Mediate the Relationship between 6′SL and Social-Emotional Development?

Since 6′SL was associated with both social development and myelination, we looked at the measured myelination regions to understand if any of them can explain the 6′SL association with ASQ using mediation analysis.

**Figure 2:**
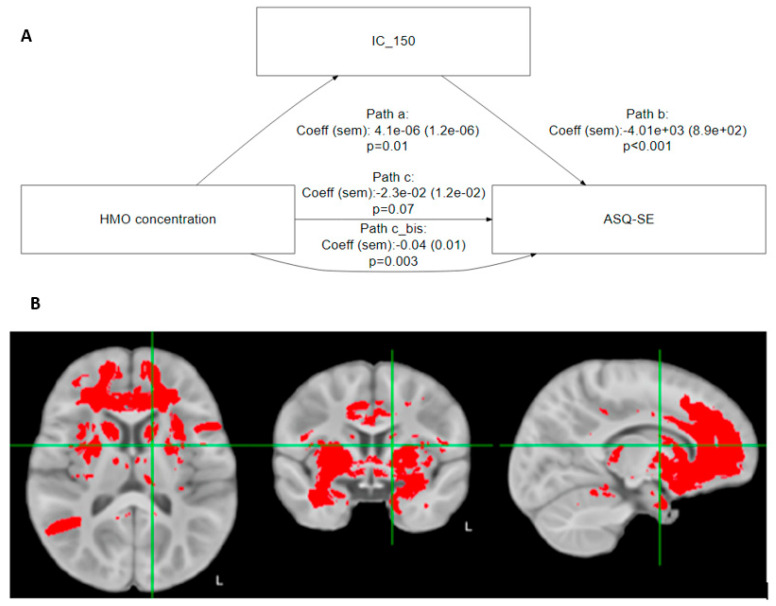
(**A**) Mediation analysis: illustrative path diagram for IC 150. Path c is the direct path from HMO to ASQ-SE:2, while path c_bis is the indirect path mediated by IC 150. (**B**) In red, the brain regions that significantly mediate the association between 6′SL and ASQ-SE:2.

Building on the preceding results, we found that brain regions commonly considered part of the ‘social brain’ [29] significantly mediated the relationship between 6′SL and ASQ-SE:2 socioemotional score (Table 4 and Figure 2A).

Based on Table 4, the association of 6′SL with ASQ-SE:2 is mediated by IC 5 (middle temporal gyrus/temporal lobe), IC 24 (inferior frontal gyrus, Broca’s area (BA 44)), IC 67 (frontal lobe, internal capsule, thalamic radiations, temporal lobe, inferior longitudinal fasciculus, frontal pole Broca’s area, Brodman area 9), IC 150 (cingulate, insula, frontal orbital cortex, thalamus, caudate nucleus), and IC 175 (frontal lobe, temporal pole).

Figure 2B shows that these regions strongly overlap with the known anatomical associations with social-emotional development and behavior.

### 3.6. Correlations between Human Milk HMO Concentration and BAYLEY-III Scores

To extend our exploration to cognitive outcome measures, we also looked for possible associations between HMOs and infant language, cognition, and motor development scores.

Bayley-III scores are summarized in Table 5.

Pairwise correlations between the concentrations of HMOs and the BAYLEY-III scores are summarized in Table 5, with significant correlations observed between language at 12 months and 3FL at V0, V1, and V2; significant correlations were found also with LNFP-II and LNFP-V at 2–5 weeks. However, none of these HMOs were significantly associated with myelination; therefore, we did not perform a mediation analysis in this case.

Evaluation of possible confounding maternal and infant parameters revealed a significant association of language with the child’s sex (functional ANOVA, *p* = 0.047), with boys having an average score of 17.0 across all visits, compared to an average score of 19.3 for girls. In addition, gestational age was positively associated with cognition, language, and motor development (Supplementary Tables S3–S5). Therefore, linear models were adjusted for sex and gestational age.

The positive association between language at 12 months and 3FL remained significant even after adjusting for sex and gestational age (Supplementary Table S6).

Our analyses were exploratory in nature and did not target, a priori, a specific HMO or a specific brain region. Rather, we adopted a data-driven approach to systematically investigate associations of all HMOs with all ICs as well as with ASQ:SE-2 and Bayley scores at multiple time points with a hypothesis that myelination may, in part, explain how HMOs may influence brain development and function.

## 4. Discussion

In the current study, we examined the association of specific individual HMOs with developmental myelination as well as motor, cognitive, language, and socio-emotional outcomes. Our analysis has revealed two main findings. First, we observed a significant inverse association of human milk 6′SL concentration in predominantly breastfed infants during the first 3 months of lactation with socio-emotional skills at 12 months, indicating a positive effect. Total sialic acid in human milk showed a similar effect. Interestingly, myelination in infants’ brain regions (which include the middle temporal gyrus, inferior frontal gyrus, Broca’s area, frontal lobe, internal capsule, thalamic radiations, temporal lobe, inferior longitudinal fasciculus, frontal pole Broca’s area, Brodman area 9, cingulate, insula, frontal orbital cortex, thalamus, caudate nucleus, frontal lobe, and temporal pole) (Figure 2) at 3 months was found to mediate the association of 6′SL with social-emotional skills observed in infants at 12 months. The second finding was significant positive associations between concentrations of specific fucosylated HMOs, namely 3FL, LNFP-II, and LNFP-V, in human milk during the first 3 months of lactation and language scores in infants at 12 months. Contrasting to the first result, no significant association was found between these fucosylated HMOs and brain myelination.

The development of social-emotional skills during infancy has implications for later life capabilities, which include emotional well-being, mental health, and decision making [36,37,38]. To date, only one study has established a link between brain growth in social brain areas (measured via myelination) and SE development (measured via ASQ:SE2), thus adding knowledge on the neural underpinnings of social behavior in infants [29]. Our results extend these findings by demonstrating associations between specific human milk components (6′SL) and overall sialic acid, brain growth (myelination), and behavior (SE). Interestingly, in terms of achievement of social skills milestones, infants begin socializing with people other than their primary and secondary caregivers at 12 months [39]. Additionally, specific brain regions such as the middle temporal gyrus, inferior frontal gyrus, Broca’s area, frontal lobe, internal capsule, thalamic radiations, temporal lobe, inferior longitudinal fasciculus, frontal pole Broca’s area, Brodman area, cingulate, insula, frontal orbital cortex, thalamus, caudate nucleus, frontal lobe, and temporal pole demonstrated myelination at 3 months, which in turn mediated the association of 6′SL concentration on social skills. The extent to which myelination mediates the effect of 6′SL is variable depending on the brain region, ranging from 30% (middle temporal gyrus/temporal lobe) to 60% (frontal lobe, temporal pole). Furthermore, the insula, orbitofrontal cortex, caudate nucleus, cingulate, and temporal lobe have been mapped as brain areas associated with social cognition and behavior [40,41,42].

6′SL is one of the most abundant sialylated HMO in human milk [43]. In addition to the 6′SL finding, we also found a significant negative correlation of total sialic acid with the ASQ-SE:2 scores at 12 months. It has been proposed as a mechanism that sialic acid serves as a conditional essential nutrient in ganglioside formation and myelination [44,45]. Yet, the exact mechanism of how dietary sialic acid may affect brain structures and function remains to be shown. Nevertheless, sialic acid has also been associated with cognition and memory in piglets [46]. While sialylated HMOs have been proposed to be the main source of sialic acid in exclusively breast-fed infants, it is interesting to note here that we consistently found 6′SL (among the most abundant sialylated HMOs) and total sialic acid correlating with the ASQ-SE:2 scores, but not other sialylated HMOs like 3′SL, DSLNT, or LST-b. For LST-c, which has a similar structural exposure to sialic acid, we also found significant associations with myelination in the social brain areas, while associations with ASQ-SE:2 were negative but not significant. Although purely speculative at this point, this suggests a certain structure–function specificity between different HMOs. Additionally, and specifically for 6′SL, the effect on ASQ-SE:2 was shown in the present study to be mediated by myelination. Sialic acid is a key component of the brain both in the form of glycoproteins and glycolipids, dubbed gangliosides when containing sialic acid. Myelination is strongly dependent on ganglioside-bound sialic acid, as demonstrated by mutant mice lacking sialic transferases St3gal2 and St3gal3, responsible for sialylation of gangliosides, that showed reduced brain myelination [47]. Our findings provide further evidence regarding sialic acid—either as a component of a specific sialylated HMO 6′SL or, overall, as a human milk component—to potentially impact myelination in early life and thereby lead to alteration of social-emotional behavior development. Previously, 6′SL in human milk has been associated with infant motor scores (at 6 and 18 months) and infant cognitive scores (at 18 months) [21,48]. To our knowledge, our study provides the first clinical observations indicating the role of the HMO 6′SL in social-emotional functions. Additionally, our study is the first to report a possible mechanism of action through the mediation by myelination.

Twelve months can be considered a pivotal age for the development of language capabilities in infants; receptive language capabilities emerge in the first 12 months of infancy due to the rapid maturation of auditory functions [49] and expressive language capabilities emerge generally from about 12 months of age [39]. Previously, two independent studies reported on associations between HMOs in human milk and language development in infancy. Jorgensen et al. [50] reported that in a Malawian mother/infant cohort, infants receiving human milk from secretor mothers with higher than median levels of either fucosylated or sialylated HMOs at 6 months of age exhibited increased vocabulary at 18 months of age. Cho et al. [20] reported an association between maternal milk concentration of 3′SL and language development specifically in a subgroup of infants from mothers with detectable A-tetrasaccharide in human milk.

Here, we identified several significant associations between specific fucosylated HMOs (3FL, LNFP II, LNFP V) and language scores in 12-month-old infants. While the concentrations of 3FL collected at 2–5 weeks, 6 weeks, and 3 months from human milk positively correlated with language scores at 12 months, the concentrations of LNFP-II and LNFP-V collected at 2–5 weeks from human milk positively correlated with language scores in 12-month-old infants. Our findings add to the results reported by Jorgensen et al. [50] associating total fucosylated HMOs with language, an effect that our results suggest being mediated mainly by 3FL, LNFP-II, and LNFP-V. Interestingly, the breastmilk concentrations of these 3 HMOs have been associated with polymorphism of the fucosyltransferase 3 (FUT3, Lewis gene), a gene coding for the enzyme responsible for the synthesis of α1-3 and α1-4 fucosylated HMOs [51,52]. For these fucosylated HMOs, we did not observe any association with myelination (Bai et al., 2018; Sánchez et al., 2021). Yet, myelination in the developing brain has been shown to predict language skills after 12 months [53]. However, in our study, we did not observe an association between myelin water fraction and any outcome measure of the Bayley scores. This could also explain the difference in terms of HMOs associated reported in the present study and others, in particular, the absence of sialylated HMO association with language compared to previous studies [20,50]. Additionally, all three studies use different tools to measure language scores in infancy: Jorgensen et al. [50] used a 100-word list based on the MacArthur–Bates Communicative Development Inventory, Cho et al. [20] used Mullen’s scale of early learning (MSEL), and our study used Bayley Scales of Infant and Toddler Development. This methodological difference could also have played a role in the lack of similarities in terms of associations observed.

While the current study has shed novel light on the role of 6′SL in myelination and social-emotional development, as well as the role of specific fucosylated HMOs (3FL, LNFP II, LNFP V) in language development, there are several limitations that future studies may need to address. Firstly, the observational nature of the current data limits us from proving causality, although the mediation by myelination represents a first step in this direction. Secondly, the current study lacks gut microbiome data that may be particularly important since different infants are known to ferment different HMOs through their developing gut microbiome. This may provide an important additional element to consider when looking for underlying mechanisms of action to explain the clinical observations. Lastly, because HMOs represent groups of key differentiating structures, and because many show covariance, it would be interesting for future studies to examine the combined effect of HMO clusters or networks on neurodevelopmental outcomes.

## 5. Conclusions

The current observational findings demonstrated an association of a specific sialylated HMO 6′SL with social-emotional skills, mediated by myelination, a key neurodevelopmental process during early life development. Additionally, this study also refines the link of fucosylated HMOs with language development, highlighting the relevance of several FUT3-dependent HMOs, namely 3FL, LNFP-II, and LNFP-V. These novel findings further strengthen our understanding of the roles of specific HMOs in neurodevelopment and associated functional outcomes, such as social-emotional function and language development that form the foundation for learning.

## 6. Patents

There is one patent filed resulting from the work reported in this manuscript.

## Figures and Tables

**Table 1:** Maternal and infant characteristics.

Ethnicity	n	%
Caucasian, White	77	71
African American, Black	8	7
Mixed race	8	7
Hispanic + Latino	7	7
Asian	3	3
Other	6	5
Income	n	%
200,000 USD or more	7	6
150,000–199,999 USD	10	9
110,000–149,999 USD	17	16
90,000–109,999 USD	9	8
70,000–89,999 USD	11	10
50,000–69,999 USD	10	9
30,000–49,999 USD	10	9
10,000–29,999 USD	10	9
Missing	25	24
Number of siblings at birth	n	%
0	41	38
1	44	40
2	14	13
3	7	6
4	1	1
5	2	2
Body measures	Mean (SD)	[Min, Max]
BMI before pregnancy	27.3 (5.8)	[19, 39.9]
Gestational Age	Mean (SD)	[Min, Max]
Weeks	39.3 (1.08)	[37.0, 41.0]
Maternal age at recruitment	Mean (SD)	[Min, Max]
Years	31 (5)	[19, 43]
Mode of delivery	n	%
Vaginal	77	72
Maternal education	n	%
Partial high school (10th or 11th grade)	2	2
High school graduate	8	7
Partial college/university	24	22
Standard college/university graduate/bachelor’s degree	30	28
Graduate degree/master’s degree/doctorate/MBA	43	40
Maternal IQ	Mean (SD)	[Min, Max]
Full-scale IQ	104 (13.6)	[80, 141]
Perceptual Reasoning Index	103 (13.4)	[75, 138]
Verbal Comprehension Index	103 (13.4)	[74, 151]
Maternal Edinburgh Post-Natal Depression Score	Mean (SD)	% above cut-off
Total score (last trimester of pregnancy, n = 76)	4.4 (4.1)	4
Total score (V1, n = 101)	4.3 (4.6)	5
Total score (V2, n = 86)	3.5 (3.5)	1
Number of feedings per day ^a^	Mean (SD)	[Min, Max]
*V0 (N = 107)*	10 (3.3)	[1, 30]
*V1 (N = 101)*	9 (2.5)	[2, 20]
*V2 (N = 86)*	8 (2.3)	[1, 14]

^a^ Question asked was: On the days the baby was fed breastmilk over the past 7 days, about how many feedings per day were breastmilk?

**Table 2:** Summary statistics for ASQ:SE-2 (Ages and Stages Questionnaire: Social-Emotional Version), by timepoint in months (mo). The statistics were calculated on all the children in the breastfed group, independently of whether they had a milk sample at the given time point.

Scale	Visit	Mean (SD)	Median [Min, Max]
ASQ:SE-2	3 mo (N = 87)	18.7 (12.7)	15 [0, 55]
	6 mo (N = 78)	18.8 (13.1)	20 [0, 75]
	12 mo (N = 61)	26.7 (18.1)	25 [0, 85]
	18 mo (N = 60)	28.1 (25.3)	20 [0, 140]
	24 mo (N = 55)	30.0 (22.3)	30 [0, 95]

**Table 3:** Adjusted linear model for ASQ-SE:2 at 12 months, with 6′SL as predictor, adjusted for maternal education.

	ASQ [12 Months]		
Predictors	Estimates	CI	*p*
(Intercept)	63.48	43.02–83.93	<0.001
VISITV1	0.39	−15.68–16.45	0.962
VISITV2	−6.87	21.89–8.15	0.368
6′SL	−0.04	−0.06–−0.02	0.001
Mother edu4	−19.76	-41.63–2.11	0.076
Mother edu5	−8.68	−27.86–10.50	0.373
Mother edu6	−18.35	−36.65–−0.06	0.049
Mother edu7	−22.34	−40.49–−4.18	0.016
VISITV1:6′SL	−0.02	−0.06–0.02	0.234
VISITV2:6′SL	−0.05	−0.11–0.02	0.147
Observations	165		
R^2^/R^2^ adjusted	0.241/0.197		

**Table 4:** Causal mediation analysis, with quasi-Bayesian confidence intervals. Significance. codes: *** *p* < 0.001, ** *p* < 0.01, * *p* < 0.05. ACME (Average Causal Mediated Effect) is the indirect effect of the HMO concentration on the ASQ that goes through the mediator (myelination).

Independent Component	Proportion Mediated	Total Effect	ACME
IC_5	0.3 ***	−0.0559 ***	−0.0166 ***
IC_24	0.3 ***	−0.058 ***	−0.0193 ***
IC_67	0.6 ***	−0.0553 ***	−0.0322 ***
IC_74	0.3 *	−0.0552	−0.0153 **
IC_150	0.4 ***	−0.0564 ***	−0.0217 ***
IC_175	0.6 ***	−0.0574 ***	−0.0274 ***

**Table 5:** Correlations between HMO concentrations (mg/L) and Bayley (after B-H correction).

Correlation	HMO [mg/L]	Outcome	Time Point Milk Sample	Time Point Bayley	N	p.adj
0.42	3FL	Language	2–5 weeks	12 months	54	0.02
0.45	3FL	Language	6 weeks	12 months	51	0.02
0.44	3FL	Language	2–5 weeks	12 months	48	0.04
0.43	LNFP-II	Language	2–5 weeks	12 months	54	0.02
0.41	LNFP-V	Language	2–5 weeks	12 months	54	0.02

## Data Availability

Datasets are available on request, without undue reservation. Deidentified individual participant data will not be made available.

## Supplementary Materials

The following supporting information can be downloaded at https://www.mdpi.com/article/10.3390/nu15214624/s1. Supplementary Figure S1. List of measured HMOs with respective structures and abbreviations. Supplementary Figure S2. (A) Flow diagram of participants; the randomized arms re not detailed here. (B) The age of the subjects at the MRI scans. Each filled circle corresponds to a study visit, horizontal lines indicate multiple longitudinal visits. Supplementary Figure S3. Depicts myelination is strongly dependent on age, even within the time window of a given study visit. Supplementary Figure S4. Positive associations between 6′SL and IC67, IC150. Supplementary Table S1. Descriptive statistics for myelin-water fraction (whole brain). Supplementary Table S2. Results from linear models for MWF as response variable and HMOs as independent variables. Only shown: N > 15, *p*.value.HMO < 0.05, R2 > 0.1. Supplementary Table S3. Effect of gestational age on Motor development. Supplementary Table S4. Effect of gestational age on Language. Supplementary Table S5. Effect of gestational age on Cognition. Supplementary Table S6. Linear model for Language at 12 months, with 3FL as independent variable, adjusted for gender and gestational age.
