# Supplementary material for: The Role of Human Milk Oligosaccharides in Myelination, Socio-Emotional and Language Development: Observational Data from Breast-Fed Infants in the United States of America
Source: Nutrients. 2023 Oct 31;15(21):4624. doi: 10.3390/nu15214624 (PMC10649431; doi:10.3390/nu15214624)
Supplement: Supplementary file 1 [file nutrients-15-04624-s001.zip › nutrients-2656224-supplementary.pdf]

Supplementary Figure S1. List of measured HMOs with respective structures and abbreviations

HMO		Abbreviation
2’-FUCOSYLLACTOSE	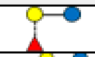	2’FL
3’-GALACTOSYLLACTOSE	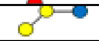	3’GL
3’-SIALYLLACTOSE	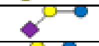	3’SL
3,2’-DIFUCOSYLLACTOSE	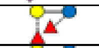	DFL
3-FUCOSYLLACTOSE	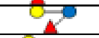	3FL
6’-GALACTOSYLLACTOSE	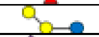	6’GL
6’-SIALYLLACTOSE	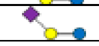	6’SL
A-TETRASACCHARIDE	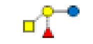	A-TETRA
DIFUCOSYLLACTO-N-HEXAOSE-a	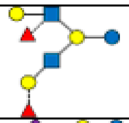	DFLNHa
DISIALYLLACTO-N-TETRAOSE	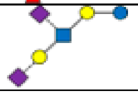	DSLNT
Unidentified hexasaccharide of composition Hex4 HexNAc2	?	Hex4 HexNAc2
LACTO-N-DIFUCOHEXAOSE-I	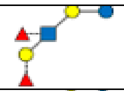	LNDFH-I
LACTO-N-FUCOPENTAOSE-I	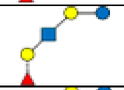	LNFP-I
LACTO-N-FUCOPENTAOSE-II	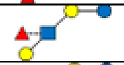	LNFP-II
LACTO-N-FUCOPENTAOSE-III	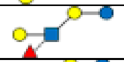	LNFP-III
LACTO-N-FUCOPENTAOSE-V	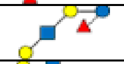	LNFP-V
LACTO-N-HEXAOSE	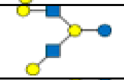	LNH
LACTO-N-NEODIFUCOHEXAOSE	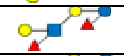	LNnDFH
LACTO-N-NEOFUCOPENTAOSE-V	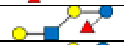	LNnFP-V
LACTO-N-NEOTETRAOSE	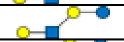	LNnT
LACTO-N-TETRAOSE	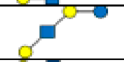	LNT
MONOFUCOSYLLACTO-N-HEXAOSE-III	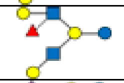	MFLNH-III
SIALYLLACTO-N-TETRAOSE-B	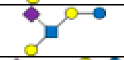	LSTb
SIALYLLACTO-N-TETRAOSE-C	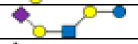	LSTc

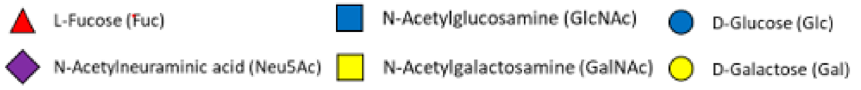

Supplementary Figure S2. A) Flow diagram of participants; the randomized arms re not detailed here. B) The age of the subjects at the MRI scans. Each filled circle corresponds to a study visit, horizontal lines indicate multiple longitudinal visits

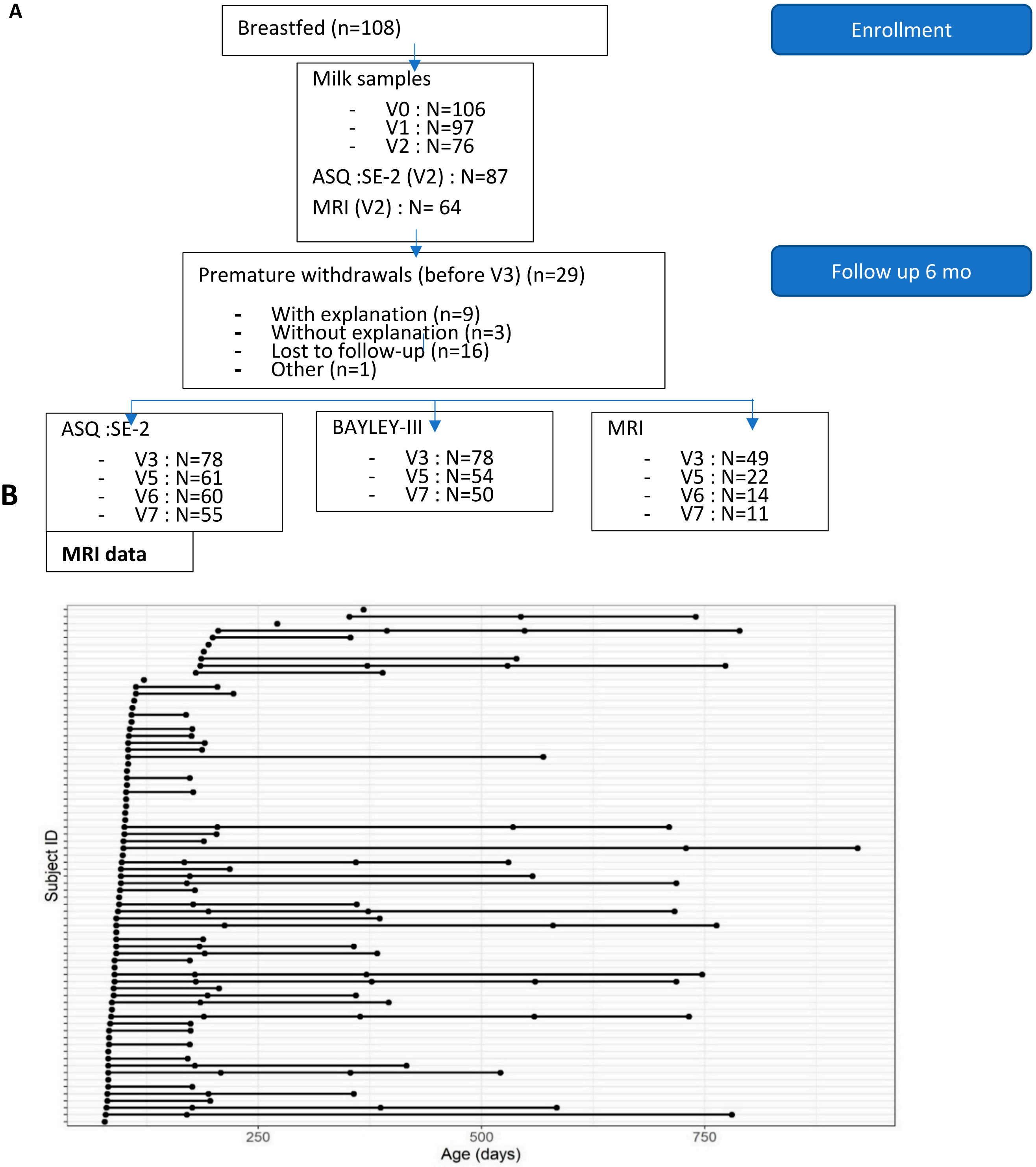

Supplementary Table S1. Descriptive statistics for myelin-water fraction (whole brain)

Visit	Mean (SD)	Median [Min, Max]	Interquartile range
3 months (N=65)	0.00247 (0.00183)	0.00207 [0, 0.00815]	(0.00103, 0.00330)
6 months (N=50)	0.0300 (0.0197)	0.0243 [0.00837, 0.120]	(0.01974, 0.03468)
12 months (N=22)	0.0800 (0.0104)	0.0795 [0.0612, 0.104]	(0.07399, 0.08455)
18 months (N=14)	0.111 (0.0246)	0.107 [0.0862, 0.175]	(0.09192, 0.12285)
24 months (N=11)	0.121 (0.0107)	0.120 [0.107, 0.136]	(0.10937, 0.12928)

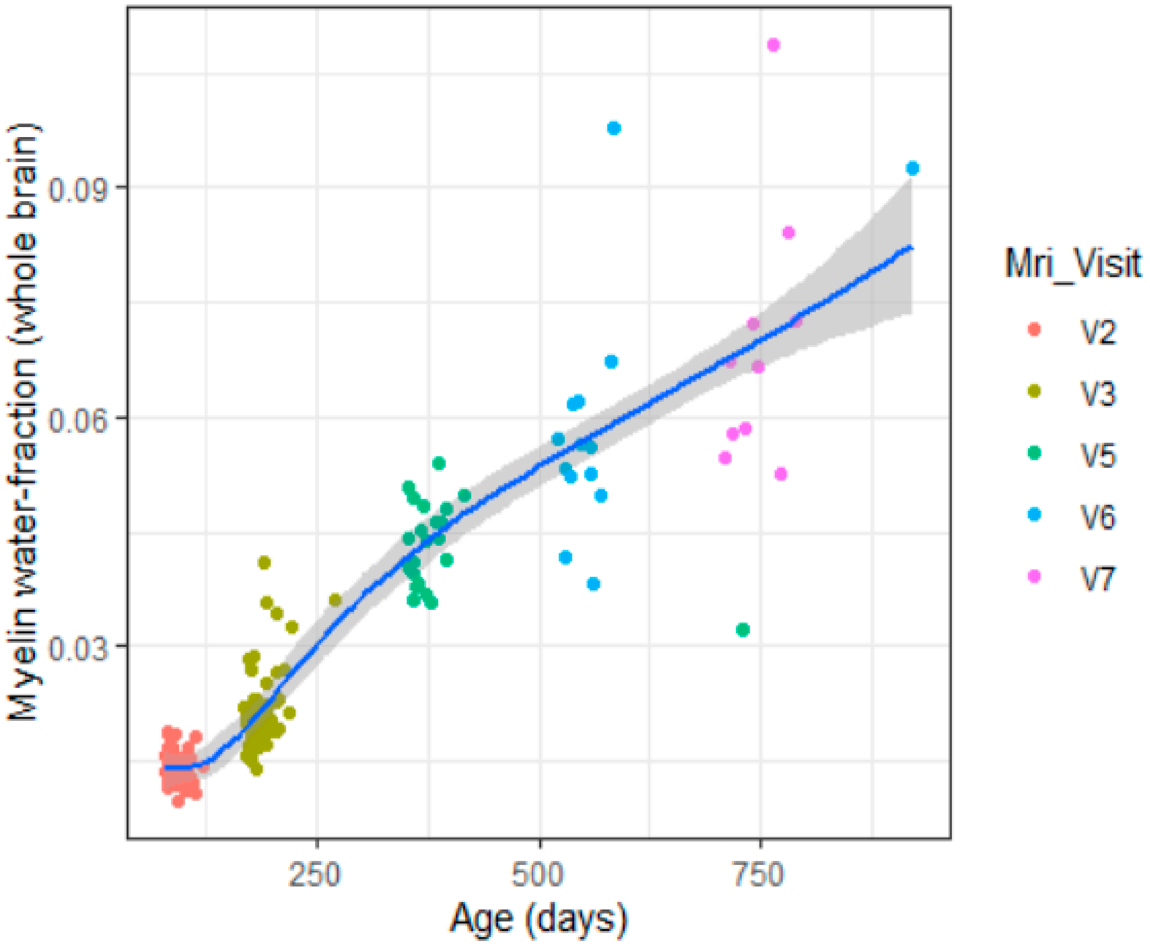

Supplementary Figure S3. Depicts myelination is strongly dependent on age, even within the time window of a given study visit.

Supplementary Table S2. Results from linear models for MWF as response variable and HMOs as independent variables. Only shown: N>15, p.value.HMO<0.05, R2>0.1

R2	mri.var	outcome	time.point. mri	time.point. HMO	p.value. HMO	coefficient. HMO	N
0.324	IC_140	DFNHA	V3	V2	0.048	-2.00E-05	41
0.228	IC_63	3'GL	V2	V1	0.004	1.20E-04	61
0.169	IC_140	3'GL	V2	V1	0.01	2.00E-04	61
0.164	IC_74	3'GL	V2	V1	0.01	1.50E-04	61
0.162	IC_147	3'GL	V2	V1	0.002	1.20E-04	61
0.137	IC_24	3'GL	V2	V1	0.005	9.00E-05	61
0.13	IC_48	3'GL	V2	V1	0.003	1.30E-04	61
0.116	IC_150	3'GL	V2	V1	0.022	9.00E-05	61
0.1	IC_132	3'GL	V2	V1	0.014	1.50E-04	61
0.185	IC_63	3'SL	V2	V0	0.02	1.00E-05	64
0.209	IC_63	6'GL	V2	V1	0.009	5.00E-05	61
0.178	IC_63	6'GL	V2	V0	0.026	4.00E-05	64
0.13	IC_74	6'GL	V2	V1	0.035	5.00E-05	61
0.125	IC_48	6'GL	V2	V1	0.004	5.00E-05	61
0.113	IC_132	6'GL	V2	V0	0.018	5.00E-05	64
0.105	IC_132	6'GL	V2	V1	0.012	6.00E-05	61
0.104	IC_150	6'GL	V2	V0	0.025	3.00E-05	64
0.162	IC_67	6'SL	V2	V0	0.009	0.00E+00	64
0.155	IC_150	6'SL	V2	V0	0.004	0.00E+00	64
0.151	IC_5	6'SL	V2	V2	0.006	1.00E-05	54
0.141	IC_140	6'SL	V2	V0	0.033	0.00E+00	64
0.138	IC_67	6'SL	V2	V2	0.007	1.00E-05	54
0.114	IC_150	6'SL	V2	V1	0.024	0.00E+00	61
0.114	IC_67	6'SL	V2	V1	0.047	1.00E-05	61

R2	mri.var	outcome	time.point.<br>mri	time.point.<br>HMO	p.value.<br>HMO	coefficient.<br>HMO	N
0.111	IC_150	6'SL	V2	V2	0.005	1.00E-05	54
0.1	IC_66	DFNHA	V2	V1	0.029	-1.00E-05	61
0.136	IC_9	DFSL	V3	V1	0.039	-1.00E-05	47
0.18	IC_74	DSNLT	V2	V0	0.004	1.00E-05	64
0.168	IC_63	DSNLT	V2	V0	0.04	0.00E+00	64
0.123	IC_74	DSNLT	V2	V1	0.044	1.00E-05	61
0.113	IC_66	DSNLT	V2	V0	0.014	1.00E-05	64
0.101	IC_48	DSNLT	V2	V0	0.009	0.00E+00	64
0.227	IC_140	LNnDFH	V2	V2	0.008	-5.00E-05	54
0.185	IC_67	LNnDFH	V2	V2	0.002	-5.00E-05	54
0.176	IC_74	LNnDFH	V2	V2	0.002	-5.00E-05	54
0.171	IC_147	LNnDFH	V2	V2	0.003	-3.00E-05	54
0.152	IC_9	LNnDFH	V3	V1	0.026	-7.00E-05	47
0.147	IC_66	LNnDFH	V2	V2	0.006	-6.00E-05	54
0.125	IC_5	LNnDFH	V2	V2	0.013	-4.00E-05	54
0.212	IC_67	LNnFP-V	V2	V1	0.001	1.00E-04	61
0.2	IC_67	LNnFP-V	V2	V0	0.002	1.30E-04	64
0.162	IC_150	LNnFP-V	V2	V1	0.004	6.00E-05	61
0.145	IC_175	LNnFP-V	V2	V1	0.002	1.00E-04	61
0.138	IC_67	LNnFP-V	V2	V2	0.007	1.20E-04	54
0.13	IC_74	LNnFP-V	V2	V1	0.035	7.00E-05	61
0.13	IC_5	LNnFP-V	V2	V1	0.017	8.00E-05	61
0.13	IC_150	LNnFP-V	V2	V2	0.003	9.00E-05	54
0.127	IC_5	LNnFP-V	V2	V2	0.012	1.20E-04	54
0.123	IC_74	LNnFP-V	V2	V0	0.036	9.00E-05	64
0.115	IC_147	LNnT	V2	V2	0.019	1.00E-05	54

R2	mri.var	outcome	time.point. mri	time.point. HMO	p.value. HMO	coefficient. HMO	N
0.13	IC_74	LNT	V2	V0	0.028	0.00E+00	64
0.164	IC_66	LSTb	V2	V2	0.004	2.00E-05	54
0.186	IC_150	LSTc	V2	V1	0.002	1.00E-05	61
0.175	IC_150	LSTc	V2	V0	0.002	1.00E-05	64
0.143	IC_67	LSTc	V2	V0	0.018	1.00E-05	64
0.139	IC_5	LSTc	V2	V2	0.009	3.00E-05	54
0.135	IC_25	LSTc	V2	V0	0.001	1.00E-05	64
0.124	IC_74	LSTc	V2	V0	0.035	1.00E-05	64
0.114	IC_150	LSTc	V2	V2	0.004	2.00E-05	54
0.106	IC_67	LSTc	V2	V2	0.017	2.00E-05	54
0.103	IC_24	LSTc	V2	V1	0.016	1.00E-05	61

Supplementary Table S3. Effect of gestational age on Motor development

	**MOTOR DEVELOPMENT**		
Predictors	*Estimates*	*CI*	*p*
(Intercept)	-8.07	-29.28 – 13.14	0.454
Gestational Age	0.68	0.14 – 1.22	**0.014**
**Random Effects**			
σ^2	12.11		
$\tau_{00\ USUBJID}$	1.44		
ICC	0.11		
$N_{USUBJID}$	78		

Observations	170
Marginal R^2 / Conditional R^2	0.041 / 0.143

Supplementary Table S4. Effect of gestational age on Language

	**LANGUAGE**		
Predictors	*Estimates*	*CI*	*p*
(Intercept)	-6.89	-29.64 – 15.86	0.551
Gestational age	0.65	0.07 – 1.22	**0.029**
**Random Effects**			
σ^2	14.30		
$\tau_{00\ USUBJID}$	1.42		
ICC	0.09		
N $_{USUBJID}$	79		
Observations	170		
Marginal R^2 / Conditional R^2	0.032 / 0.119		

Supplementary Table S5. Effect of gestational age on Cognition

	Cognition		
Predictors	*Estimates*	*CI*	*p*
(Intercept)	-4.06	-16.37 – 8.25	0.516
Gestational age	0.36	0.05 – 0.67	**0.024**
**Random Effects**			
σ^2	3.54		
$\tau_{00\ USUBJID}$	0.69		
ICC	0.16		
$N_{USUBJID}$	79		
Observations	162		
Marginal R^2 / Conditional R^2	0.038 / 0.195		

Supplementary Table S6. Linear model for Language at 12 months, with 3FL as independent variable, adjusted for gender and gestational age

term	estimate	std.error	p.value
(Intercept)	-28.45	9.87	0.00
3FL	18.87	6.60	0.00
Gestational Age	1.16	0.25	0.00
SEX: Male	-1.57	0.59	0.01
VISIT: V1	-0.23	1.03	0.83
VISIT: V2	-0.31	1.07	0.77
3FL:VISIT V1	-3.92	8.39	0.64
3FL:VISIT V2	-8.67	7.58	0.25

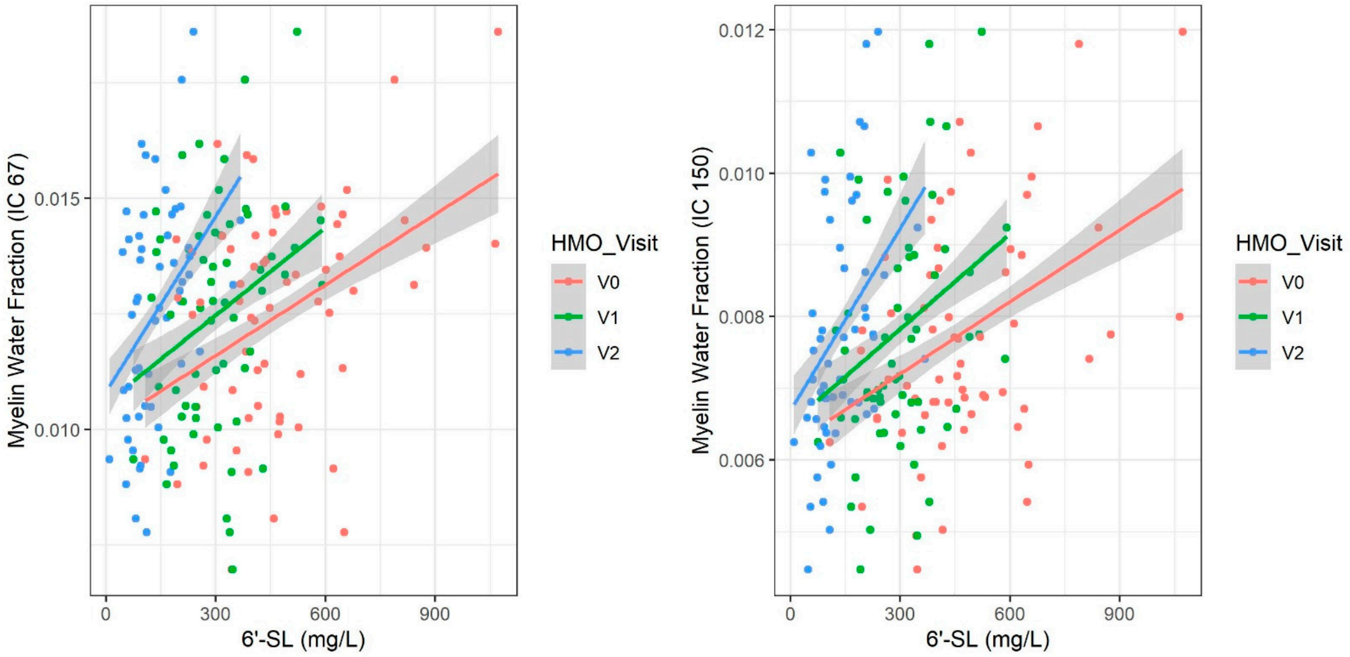

Supplementary Figure S4 Positive associations between 6'SL and IC67, IC150
